# Superheating Control of ORC Systems via Minimum (*h*,*φ*)-Entropy Control

**DOI:** 10.3390/e24040513

**Published:** 2022-04-06

**Authors:** Jianhua Zhang, Jinzhu Pu, Mingming Lin, Qianxiong Ma

**Affiliations:** 1 State Key Laboratory of Alternate Electrical Power System with Renewable Energy Sources, North China Electric Power University, Beijing 102206, China; zjh@ncepu.edu.cn; 2 School of Control and Computer Engineering, North China Electric Power University, Beijing 102206, China; 1162127005@ncepu.edu.cn (J.P.); qianxiongma@163.com (Q.M.); 3 School of Automation and Electronic Engineering, Qingdao University of Science and Technology, Qingdao 266061, China

**Keywords:** Organic Rankine Cycle, superheating, minimum (*h*,*φ*)-entropy control, non-Gaussian

## Abstract

The Organic Rankine Cycle (ORC) is one kind of appropriate energy recovery techniques for low grade heat sources. Since the mass flow rate and the inlet temperature of heat sources usually experience non-Gaussian fluctuations, a conventional linear quadratic performance criterion cannot characterize the system uncertainties adequately. This paper proposes a new model free control strategy which applies the (*h*,*φ*)-entropy criterion to decrease the randomness of controlled ORC systems. In order to calculate the (*h*,*φ*)-entropy, the kernel density estimation (KDE) algorithm is used to estimate the probability density function (PDF) of the tracking error. By minimizing the performance criterion mainly consisting of (*h*,*φ*)-entropy, a new control algorithm for ORC systems is obtained. The stability of the proposed control system is analyzed. The simulation results show that the ORC system under the proposed control method has smaller standard deviation (STD) and mean squared error (MSE), and reveals less randomness than those of the traditional PID control algorithm.

## 1. Introduction

As a promising technique for low grade heat recovery particularly in small-scale systems, Organic Rankine Cycle (ORC) technology has advantages in the aspects of wide application for various heat sources, strong part-load performance and simplicity in structure. It is also suitable for transient heat sources, such as waste heat from engines, solar energy, intermittent industrial waste heat, and so on [1,2,3]. These transient heat sources are usually under non-Gaussian circumstances. The mass flow rate and inlet temperature of heat sources in ORCs are not necessarily Gaussian. Moreover, the nonlinearities in ORCs could lead to non-Gaussian randomness, even if heat sources follow Gaussian distribution.

Superheating control is of great significance for ORC systems on security and economy [4]. Superheating of the ORC systems should be kept positive in case of liquefaction of the working fluid, which may cause turbine blade damage [5]. For most dry working fluids, superheating should be kept low enough to ensure high system efficiency. However, low superheating may disappear when heat sources experience large fluctuations during practical operation. Thus, the random nature of heat sources brings severe challenges to the ORC superheating control.

Tremendous research on superheating control of the ORC system has been conducted: recent reviews on control strategies, working fluid selection and dynamic modeling of the ORC system can be looked over in [6,7,8]. The PID control method has extensive application in ORC systems. Three control methods based on PID controller were proposed in [9]. The pump speed and the expander speed were the two freedom degrees to control the evaporating temperature and the superheating of the ORC system. Reference [10] proposed an adaptive PID control algorithm with feed-forward compensation and received satisfying control performance. Reference [11] applied an integrated control algorithm based on PID with a feed-forward control scheme for control performance enhancement of the ORC system. The optimal control law of the ORC system was obtained by solving a constrained optimization problem based on an established model. Nevertheless, these control algorithms may not receive satisfying control performance for ORC systems with fluctuating heat sources by virtue of the limitation of the disturbance rejection.

In order to enhance the disturbance rejection capability of the ORC control system with transient heat sources, tremendous research on the advanced control strategies was conducted recently. Reference [12] proposed a nonlinear model predictive control with approximate NMPC solutions investigated for computation reduction for the ORC waste heat recovery system. The proposed control algorithm outperformed the PI based controller with a feedforward control scheme. A two-layer MPC based control method was proposed in [13] and received satisfying control performance. Reference [14] applied a multiple model predictive control method to ORC systems. The local dynamics of the ORC system were presented by different model structures while keeping the same complexity of the optimization problem.

Yao et al. [15] proposed a dual-mode fast dynamic matrix control (FDMC) algorithm for the ORC system. Compared with MPC, the proposed control method could increase the speed of calculating significantly with control performance guaranteed.

However, the aforementioned research hardly coped with the stochastic disturbances from heat sources in ORC systems, not to mention that the temperature and mass flow rate of the heat sources are commonly non-Gaussian; it calls for a stochastic control framework.

Minimum error entropy (MEE) was employed for stochastic control in many research works [16,17,18,19,20]. Shannon entropy is commonly used in MEE-based stochastic control, and Renyi entropy is a generalization of Shannon entropy. When the order of Renyi entropy approaches 1, Renyi entropy will reduce to Shannon entropy. $\left(h,\varphi \right)$-entropy is the most generalized definition of entropy [21,22,23]. Numerical examples have indicated that the $\left(h,\varphi \right)$-entropy criterion could achieve satisfactory error distribution [24]. Thus, $\left(h,\varphi \right)$-entropy is employed as a performance index to attenuate the randomness of ORC systems and achieve accurate tracking of superheating. Accordingly, in this paper, a performance criterion that mainly consists of error $\left(h,\varphi \right)$-entropy is established, then a model-free control algorithm for the ORC systems can be obtained by minimizing the performance criterion, and the comparative simulation results testify its effectiveness. Furthermore, the stability analysis of the superheating control system is carried out. The contributions of this work are summarized as follows:

1. A model-free minimum (*h,φ*)-entropy method is proposed for superheating control of ORC systems, and (*h,φ*)-entropy is applied to characterize the randomness of the system;
2. The stability analysis of the proposed control is given;
3. A simulation example is used to testify the effectiveness of the proposed control strategy.

The remainder of the paper goes as follows. Section 2 gives an introduction of the ORC system and its mathematical model. Section 3 provides a generalized $\left(h,\varphi \right)$-entropy criterion and its estimation by the kernel density estimation method; the control algorithm for superheating control systems is then obtained by minimizing the criterion. The effectiveness of the proposed control algorithm for the ORC system is demonstrated by a simulation in Section 4. Finally, Section 5 concludes this paper.

## 2. System Description

**Figure 1:**
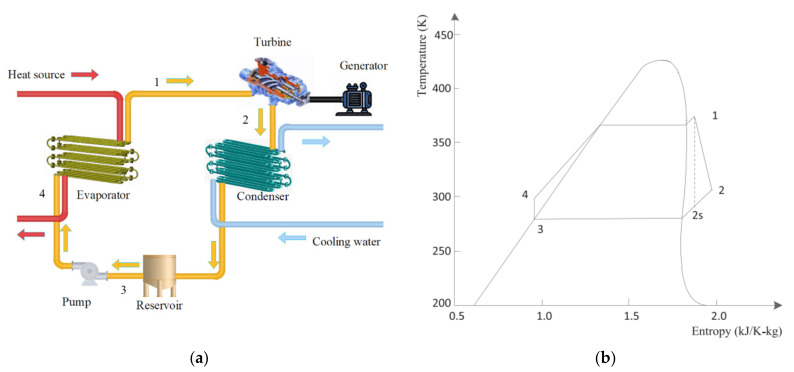
(**a**) Schematic diagram of an ORC system, and (**b**) T-s thermodynamic diagram for the ORC system.

In this section, the considered ORC system with a transient heat source is shown in Figure 1a, and the corresponding T-s thermodynamic diagram is shown in Figure 1b. Despite the superheating control method proposed in this work being model free, the model of the ORC system is still established for the stability analysis.

As shown in Figure 1a, the working fluid of the ORC system is heated to superheated vapor by the evaporator and then sent to the turbine for power generation; the exhaust vapor, after doing work, is condensed to liquid by the condenser and then conducted to a reservoir. The working fluid in the reservoir is next pumped to the evaporator, and then the whole cycle of the ORC system is completed. The working fluid of the investigated ORC system is chosen to be R245fa considering its high efficiency and appropriate thermophysical properties for the ORC systems with low grade heat sources [25,26]. Notice that the superheating of the vapor heated by the evaporator should be kept within a proper range for the safety and efficiency of the ORC system.

The model of the ORC system is established for the stability analysis hereafter.

### 2.1. Evaporator and Condenser

**Figure 2:**
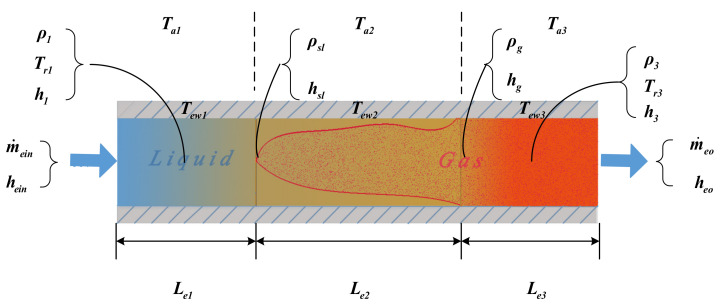
MB model of an evaporator.

The mathematical model of the evaporator is established by the moving boundary (MB) method in which the evaporator is divided into three regions as shown in Figure 2 [27]. Then the energy and mass balance equations of each region can be represented as Equations (1)–(3) by the lumped parameter method.

(1) $$\int_{0}^{L_{i}}\frac{\partial A\rho }{\partial t}dl+\int_{0}^{L_{i}}\frac{\partial {\dot{m}}_{e}}{\partial l}dl=0$$

where $L_{i}$ stands for length of divided region and $i\in \left\{1,2,3\right\}$, A denotes the cross sectional area of the evaporator, ρ is the density, t is time, l denotes length coordinate and ${\dot{m}}_{e}$ stands for the mass flow rate.

The energy balance equation for each divided region can be represented as

(2) $$\int_{0}^{L_{i}}\frac{\partial \left(\rho Ah-AP\right)}{\partial t}dl+\int_{0}^{L_{i}}\frac{\partial {\dot{m}}_{e}h}{\partial l}dl=\int_{0}^{L_{i}}\pi Dr_{in}\eta_{in}\left(T_{ew}-T_{r}\right)dl$$

where h stands for the enthalpy of the working fluid, P is the pressure, $Dr_{in}$ stands for the diameter of the inner tube, $\eta_{in}$ denotes the heat transfer coefficient, $T_{ew}$ stands for the temperature of the wall and $T_{r}$ denotes the temperature of the working fluid.

The energy balance equation of the tube wall can be described as

(3) $${\left(C_{p}\rho A\right)}_{w}\frac{\partial T_{ew}}{\partial t}=\pi Dr_{in}\eta_{in}\left(T_{r}-T_{ew}\right)+\pi Dr_{o}\eta_{o}\left(T_{a}-T_{ew}\right)$$

where $C_{p}$ stands for heat capacity, w stands for the wall of evaporator, $Dr_{o}$ is the diameter of outer tube, $\eta_{o}$ stands for heat transfer and $T_{a}$ denotes the temperature of the heat source.

Then the model of evaporator can be obtained by integrating Equations (1)–(3) over the three divided regions

(4) $${\dot{x}}_{e}=D_{e}{}^{-1}f_{e}\left(x_{e},u_{e}\right)$$

where $x_{e}={\left[L_{e}{}_{1},L_{e}{}_{2},P_{e},h_{e}{}_{o},T_{e}{}_{w1},T_{e}{}_{w2},T_{e}{}_{w3}\right]}^{T}$ stands for the state variable and $u_{e}={\left[{\dot{m}}_{ein},h_{e}{}_{in},{\dot{m}}_{eo},{\dot{m}}_{a},T_{a}\right]}^{T}$ denotes the input vector.

The model of the condenser can be obtained by the same way as evaporator [28,29]

(5) $${\dot{x}}_{c}=D_{c}{}^{-1}f_{c}\left(x_{c},u_{c}\right)$$

where $x_{c}={\left[L_{c}{}_{1},L_{c}{}_{2},P_{c},h_{c}{}_{o},T_{c}{}_{w1},T_{c}{}_{w2},T_{c}{}_{w3}\right]}^{T}$ stands for the state variable, and $u_{c}={\left[{\dot{m}}_{cin},h_{c}{}_{in},{\dot{m}}_{co},{\dot{m}}_{c},T_{c}\right]}^{T}$ stands for the input of the condenser.

### 2.2. Turbine

The model of the turbine can be expressed by a semi empirical form shown as

(6) $${\dot{m}}_{\exp}=\frac{ff\cdot V_{\mathrm{ss},\exp}\cdot NN_{\exp}}{60\cdot \upsilon_{i,\exp}}$$

where ${\dot{m}}_{\exp}$ stands for the mass flow rate, ff is the filling factor, $V_{\mathrm{ss},\exp}$ stands for the swept volume, $NN_{\exp}$ is the rotating speed and $\upsilon_{i,\exp}$ denotes the specific volume.

### 2.3. Pump

The model of the pump can be achieved by the similarity principle expressed as

(7) $${\dot{m}}_{pp}=\frac{NN_{pp}}{NN_{ra,pp}}{\dot{m}}_{ra,pp}$$

where ${\dot{m}}_{pp}$ stands for the mass flow rate, $NN_{pp}$ and $NN_{ra,pp}$ stand for the rotating speed and rated speed of pump, respectively, and ${\dot{m}}_{ra,pp}$ denotes the rated mass flow rate.

### 2.4. Overall Model

In this work, superheating of the ORC system is the controlled variable (CV), which is mainly influenced by the speed of pump, then the overall model of the ORC system can be obtained by integrating Equations (1)–(7) expressed as

(8) $$\left\{\begin{array}{ll} \dot{x} & =g\left(x_{},u_{},\omega_{1},\omega_{2}\right)\\ y_{} & =f\left(x_{},u_{}\right) \end{array}\right.$$

where $u_{}$ stands for the manipulated variable (MV), that is pump speed, y denotes the superheating, $x={\left[L_{e}{}_{1},L_{e}{}_{2},P_{e},h_{e}{}_{o},T_{e}{}_{w1},T_{e}{}_{w2},T_{e}{}_{w3},L_{c}{}_{1},L_{c}{}_{2},P_{c},h_{c}{}_{o},T_{c}{}_{w1},T_{c}{}_{w2},T_{c}{}_{w3}\right]}^{T}$ stands for the state variable, $\omega_{1}$ stands for the mass flow rate ${\dot{m}}_{a}$ and $\omega_{2}$ denotes the inlet temperature $T_{a}$ of the heat source. $\omega_{1}$ and $\omega_{2}$, treated as disturbances of the ORC system, are mutual independent and follow non-Gaussian distribution.

Based on the overall model of the ORC system (8), the discrete-time state-space equation in nominal conditions can then be derived as

(9) $$\left\{\begin{array}{ll} x_{k+1} & =G\left(x_{k},u_{k},\omega_{1k},\omega_{2k}\right)\\ y_{k} & =F\left(x_{k},u_{k}\right) \end{array}\right.$$

where k denotes the sampling time.

## 3. Minimum (*h*,*φ*)-Entropy Control Algorithm for the ORC System

This section may be divided by subheadings. It should provide a concise and precise description of the experimental results, their interpretation, as well as the experimental conclusions that can be drawn.

**Figure 3:**
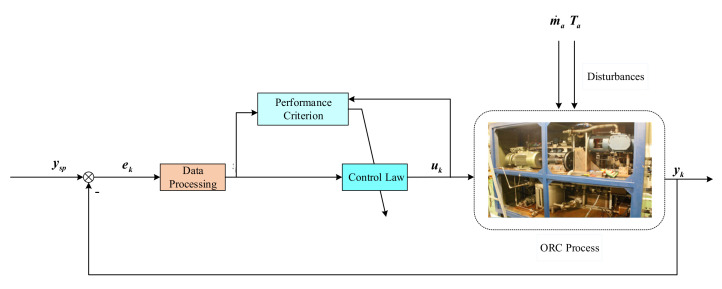
Superheating control system.

Since the ORC systems are disturbed by the fluctuations of the mass flow rate ${\dot{m}}_{a}$ and inlet temperature $T_{a}$ of the highly transient heat source, the superheating variable *y* is commonly non-Gaussian. For the safety and efficiency of ORC systems, the control objective is to minimize the randomness of tracking error $e=y_{sp}-y$, where $y_{sp}$ is the set-point of superheating. The schematic diagram of the proposed superheating control system is illustrated in Figure 3.

### 3.1. (h,φ)-Entropy and Kernel Density Estimation

As a unification of entropy measures [24], $\left(h,\varphi \right)$-entropy is employed for the superheating control system of ORC processes.

Since the superheating *y* is non-Gaussian, the tracking error is therefore non-Gaussian, and the randomness of the tracking error can then be measured by $\left(h,\varphi \right)$-entropy expressed as

(10) $$H_{\phi }^{h}(e)=h\left(\int_{-\infty }^{\infty }\phi \left[p_{k}\left(e\right)\right]de\right)$$

where either ϕ:[0,∞)→R is concave and h:R→R is increasing, or ϕ:[0,∞)→R is convex and h:R→R is decreasing. $p_{k}(\cdot )$ stands for the probability density function (PDF) at time *k*.

By using the kernel density estimation (KDE) method, the estimation of the PDF of tracking error $p_{k}\left(e\right)$ can be obtained expressed as

(11) $${\hat{p}}_{k}\left(e\right)=\frac{1}{N}\sum_{j=1}^{N}G_{\sigma }\left(e-e_{j}\right)$$

where G stands for the kernel function, σ denotes the bandwidth of the kernel function, and N is window width.

Afterwards, the estimation of tracking error $\left(h,\varphi \right)$-entropy can be calculated through the multiple imputation method shown as

(12) $${\hat{H}}_{\phi }^{h}\left(e\right)=h\left(\frac{1}{N}\sum_{i=1}^{N}\phi \left[\frac{1}{N}\sum_{j=1}^{N}G_{\sigma }\left(e_{i}-e_{j}\right)\right]\right)$$

Notice that the $\left(h,\varphi \right)$-entropy is equal to the Shannon entropy when $h\left(x\right)=x$ and $\varphi \left(x\right)=-x\log x$, and it also equals to the α-order Renyi entropy when $h\left(x\right)={\left(1-\alpha \right)}^{-1}\log x$ and $\varphi \left(x\right)=x^{\alpha }$.

Therefore, the following performance criterion is used to obtain the control law

(13) $$J_{k}=Q{\hat{H}}_{\phi }^{h}\left(e_{k}\right)+\frac{1}{2}\Delta u_{k}{}^{T}R_{}\Delta u_{k}$$

where $Q_{}$ and $R_{}$ denote the weights, ${\hat{H}}_{\phi }^{h}\left(e_{k}\right)$ is the estimated $\left(h,\varphi \right)$-entropy of tracking error *e* at time *k*, and $\Delta u_{k}=u_{k}-u_{k-1}$.

### 3.2. Control Algorithm

The superheating control law for the ORC system can be obtained by minimizing the performance criterion (13) as

(14) $$u_{k}*=\arg\;\underset{u_{k}}{\min}J_{k}=\arg\underset{u_{k}}{\min}\left(Q{\hat{H}}_{\phi }^{h}\left(e_{k}\right)+\frac{1}{2}u_{k}{}^{T}R_{}u_{k}\right)$$

Define $\tilde{\psi }(u_{k})={\hat{H}}_{\phi }^{h}\left(e_{k}\right)$, and the following approximate equation holds

(15) $$\tilde{\psi }(u_{k})\approx {\tilde{\psi }}_{k0}+{\tilde{\psi }}_{k1}\Delta u_{k}+\frac{1}{2}\Delta u_{k}{}^{T}{\tilde{\psi }}_{k2}\Delta u_{k}$$

where

$${\tilde{\psi }}_{k0}=\tilde{\psi }(u_{k})\left|{}_{u_{k}=u_{k-1}}\right.,$$

$${\tilde{\psi }}_{k1}={\left.\frac{\partial \tilde{\psi }(u_{k})}{\partial u_{k}}\right|}_{u_{k}=u_{k-1}},$$

$${\tilde{\psi }}_{k2}={\left.\frac{\partial {\tilde{\psi }}^{2}(u_{k})}{\partial u_{k}{}^{2}}\right|}_{u_{k}=u_{k-1}}.$$

The optimal $\Delta u_{k}$ should be calculated from

(16) $$\frac{\partial J_{k}}{\partial \Delta u_{k}}=0$$

By substituting Equations (14) and (15) to Equation (16), the following equation can be deduced

(17) $$\begin{array}{ll} \frac{\partial J_{k}}{\partial \Delta u_{k}} & =\frac{\partial \left(Q\left({\tilde{\psi }}_{k0}+{\tilde{\psi }}_{k1}\Delta u_{k}+\frac{1}{2}\Delta u_{k}{}^{T}{\tilde{\psi }}_{k2}\Delta u_{k}\right)+\frac{1}{2}\Delta u_{k}{}^{T}R\Delta u_{k}\right)}{\partial \Delta u_{k}}\\ & =Q\left({\tilde{\psi }}_{k1}+{\tilde{\psi }}_{k2}\Delta u_{k}\right)+R\Delta u_{k}\\ & =0 \end{array}$$

Then the optimal $\Delta u_{k}$ can be calculated as

(18) $$\Delta u_{k}*=-{\left(Q{\tilde{\psi }}_{k2}+R\right)}^{-1}Q{\tilde{\psi }}_{k1}$$

satisfying $\left(Q{\tilde{\psi }}_{k2}+R\right)>0$.

Therefore, the optimal control strategy for the ORC superheating control system can be obtained as

(19) $$u_{k}*=u_{k-1}+\Delta u_{k}*=u_{k-1}-{\left(Q{\tilde{\psi }}_{k2}+R\right)}^{-1}Q{\tilde{\psi }}_{k1}$$

The procedure of the proposed minimum $\left(h,\phi \right)$-entropy superheating control algorithm of ORC systems can be summarized by the pseudo code in Algorithm 1.
**Algorithm 1:** Minimum $\left(h,\phi \right)$-entropy superheating control algorithm of ORC systems**Input**: Simulation time T, simulation period $T_{s}$, window width N, weights of performance criterion $Q_{}$ and $R_{}$. **Initialization**: Input u, state variable of ORC x, sampling data set of tracking error e.**Steps:** **for**k←1 to $T/T_{s}$**do**  Run the ORC system, collect the sampling data of *y* and e   Update the time sampling data set of tracking error   Estimate the PDF of tracking error by the KDE method  Calculate the $\left(h,\phi \right)$-entropy of tracking error by Equation (12)  Obtain ${\tilde{\psi }}_{k0}$, ${\tilde{\psi }}_{k1}$ and ${\tilde{\psi }}_{k2}$   Calculate the optimal control input *u* by Equation (19) **end for** **Final****Return** the PDF of tracking error 

### 3.3. Stability Analysis

The stability analysis of the investigated ORC superheating control system is carried out by the statistical linearization method [30] in this section. By substituting the optimal control law (19) into the ORC system (9), the closed-loop control system can be expressed as

(20) $$\begin{array}{ll} x_{k+1} & =G\left(x_{k},u_{k-1}-{\left(Q{\tilde{\psi }}_{k2}+R\right)}^{-1}Q{\tilde{\psi }}_{k1},\omega_{k}\right)\\ & =\psi \left(x_{k},u_{k-1},Q,R,\omega_{k}\right) \end{array}$$

where $\psi \left(\cdot \right)={\left[\psi_{1}\left(\cdot \right),\cdots ,\psi_{n}\left(\cdot \right)\right]}^{T}$ is a vector function and $\omega_{k}={\left[\omega_{1k},\omega_{2k}\right]}^{T}$, then the linearization of $\psi \left(x_{k},u_{k-1},Q,R,\omega_{k}\right)$ can be obtained by statistical linearization method [31], shown as follows:

(21) $$\begin{array}{ll} \psi^{*}\left(x_{k},u_{k-1},Q,R,\omega_{k}\right)= & \psi_{0}\left(Em_{k},\theta_{k},u_{k-1},Q,R\right)\\ & +K_{\psi }\left(Em_{k},\theta_{k},u_{k-1},Q,R\right)X_{k}{}^{0} \end{array}$$

(22) $$\left[\begin{array}{l} \psi_{1}{}^{*}\\ \psi_{2}{}^{*}\\ \vdots \\ \psi_{n}{}^{*} \end{array}\right]=\left[\begin{array}{l} \psi_{10}\\ \psi_{20}\\ \vdots \\ \psi_{n0} \end{array}\right]+\left[\begin{array}{cccccc} k_{11} & \cdots & k_{1n} & k_{1(n+1)} & \cdots & k_{1(n+q_{1})}\\ k_{21} & \cdots & k_{2n} & k_{2(n+1)} & \cdots & k_{2(n+q_{1})}\\ \vdots & \ddots & \vdots & \vdots & \ddots & \vdots \\ k_{n1} & \cdots & k_{nn} & k_{n(n+1)} & \cdots & k_{n(n+q_{1})} \end{array}\right]X_{k}{}^{0}$$

where $X_{k}=\left[x_{k}{}^{T},\omega_{k}{}^{T}\right]$ and $X_{k}{}^{0}=X_{k}-Em_{k}$. $Em_{k}=E\left[X_{k}\right]$ and $\theta_{k}=E\left[{\left(X_{k}{}^{0}\right)}^{T}X_{k}{}^{0}\right]$ are the mathematical expectation and covariance matrix of random variable $X_{k}$, respectively. $\psi_{0}$ and $K_{\phi }\left(\cdot \right)=k_{ij}\left(\cdot \right)$ are the statistical feature vector and amplification coefficient matrix of the nonlinear function $\psi_{}$, respectively, which can be obtained by minimizing the following mean square approximate error criterion:

(23) $$\bar{\xi }=E\left[{\left(\psi -\psi_{0}-K_{\psi }X_{k}{}^{0}\right)}^{T}\left(\psi -\psi_{0}-K_{\psi }X_{k}{}^{0}\right)\right]$$

(24) $$\begin{array}{ll} \psi_{0}\left(x_{k},u_{k-1},Q,R,\omega_{k}\right) & =E\left[\psi \left(x_{k},u_{k-1},Q,R,\omega_{k}\right)\right]\\ & =\int_{\Omega_{X_{k}}}\psi \left(x_{k},u_{k-1},Q,R,\omega_{k}\right)p_{X_{k}}\left(\tau \right)d\tau \end{array}$$

where $p_{X_{k}}\left(\tau \right)$ is the joint PDF of $X_{k}$ which can be achieved by probability theory [27]

$$k_{ij}\left(\cdot \right)=\sum_{l=1}^{n+q_{1}}{\left(-1\right)}^{j-1}\frac{D_{j}^{i}}{D}\theta_{i}l,\;\;\;\;\;\;i=1,2,\cdots (n+q_{1})$$

(25) $$D=\left|\begin{array}{cccc} \theta_{11} & \theta_{12} & \cdots & \theta_{1(n+q_{1})}\\ \theta_{21} & \theta_{22} & \cdots & \theta_{2(n+q_{1})}\\ \vdots & \vdots & \ddots & \vdots \\ \theta_{(n+q_{1})1} & \theta_{(n+q_{1})2} & \cdots & \theta_{\left(n+q_{1}\right)}{}_{\left(n+q_{1}\right)} \end{array}\right|$$

where $D_{j}^{i}$ is the complement minor of the element in the $j^{th}$ row and $l^{th}$ column of matrix D and

(26) $$\theta_{ij}=\left[X_{i}^{0},X_{j,k}^{0}\right]=\int_{\Omega_{X_{k}}}\left(\tau_{i}-Em_{X_{ik}}\right)\left(\tau_{j}-Em_{X_{jk}}\right)d\tau$$

(27) $$\begin{array}{ll} \theta_{\psi_{i}l} & =E\left[\psi_{i}\left(x_{k},u_{k-1},Q,R\right)X_{lk}^{0}\right]\\ & =\int_{\Omega_{X_{k}}}\psi_{i}\left(x_{k},u_{k-1},Q,R\right)\left(\tau_{l}-Em_{X_{lk}^{}}\right)\gamma_{X_{k}}\left(\tau \right)d\tau \end{array}$$

Substituting (22) into (21), the statistical linearization system can be expressed as

(28) $$\begin{array}{ll} x_{k+1}= & K_{\psi x}\left(Em_{k},\theta_{k},u_{k-1},Q,R\right)+\psi_{0}\left(Em_{k},\theta_{k},u_{k-1},Q,R\right)\\ & +K_{\psi x}\left(Em_{k},\theta_{k},u_{k-1},Q,R\right)Em_{xk}\\ & +K_{\psi x}\left(Em_{k},\theta_{k},u_{k-1},Q,R\right)\left(\omega_{k}-Em_{\omega_{k}}\right) \end{array}$$

where $Em_{x_{k}}=E\left(x_{k}\right)$ and $Em_{\omega_{k}}=E\left(\omega_{k}\right)$.

Define

(29) $$\begin{array}{ll} \varsigma_{k}= & \psi_{0}\left(Em_{k},\theta_{k},u_{k-1},Q,R\right)-K_{\psi x}\left(Em_{k},\theta_{k},u_{k-1},Q,R\right)Em_{xk}\\ & +K_{\psi \omega }\left(Em_{k},\theta_{k},u_{k-1},Q,R\right)\left(\omega_{k}-Em_{\omega_{k}}\right) \end{array}$$

Then, the statistical linearization system (28) can be reformulated as

(30) $$x_{k+1}=K_{\psi x}\left(Em_{k},\theta_{k},u_{k-1},Q,R\right)X_{k}+\varsigma_{k}$$

$\varsigma_{k}$ is bounded because $\omega_{k}$ is bounded. Hence, the linearized closed loop control system (28) is stable if $x_{k}$ is bounded. Therefore, the convergence condition of the investigated superheating control system can be expressed as

(31) $$\left\|K_{\psi x}\left(Em_{k},\theta_{k},u_{k-1},Q,R\right)\right\|<1$$

## 4. Simulation Studies

**Figure 4:**
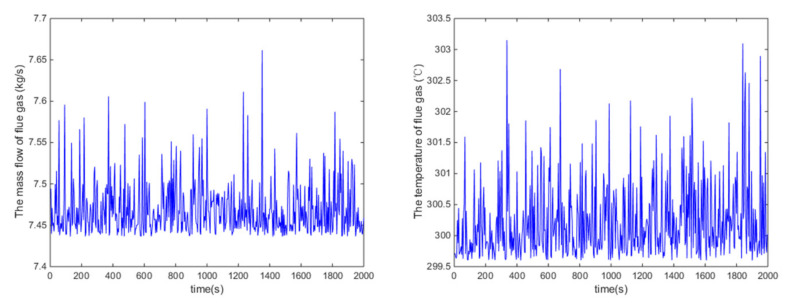
Mass flow rate and inlet temperature of the heat source over time.

**Figure 5:**
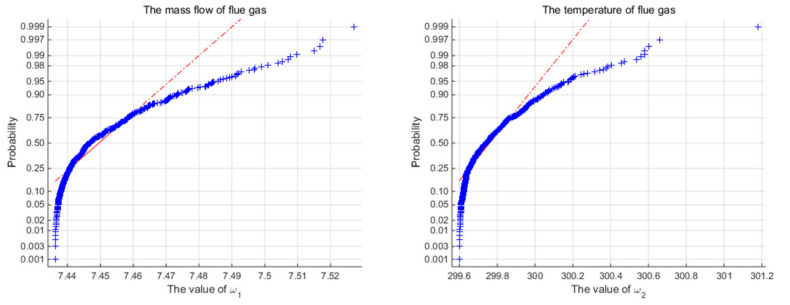
The normal probability plots of the inlet temperature and the mass flow rate.

The effectiveness of the proposed control algorithm for the ORC system is testified in this section. Superheating is the controlled variable, and the pump speed is the manipulated variable (MV) in the simulations. The variations of the mass flow rate and inlet temperature of the heat source over time in the set-point tracking test are illustrated in Figure 4. As shown in Figure 5, the sample data of mass flow rate and inlet temperature of the heat source are illustrated as symbol ‘+’ in the normal probability plots, and they do not follow a straight line, which means that they are characterized to be non-Gaussian.

Two simulation tests are carried out to testify the set-point tracking ability and disturbance rejection ability of the proposed control algorithm for the ORC system. As a contrast, the performance of the PID control algorithm is also tested. In the simulations, as one of the specific $\left(h,\varphi \right)$-entropy, $\phi \left(x\right)=x^{r/m}$, $h\left(x\right)={\left(m\left(m-r\right)\right)}^{-1}\log x$ are chosen, where m=0.5 and r=1. With the parameters tuned by Matlab software, the transfer function of the PID controller is $G_{\mathrm{PID}}(s)=30\times \left[1+4/\left(15s\right)+3s/4\right]$.

### 4.1. Tracking Ability Test

In this test, to testify the set-point tracking ability of the proposed control algorithm, a 0.5 °C step descent on the set-point of superheating occurs at 100 s. The simulation results of the PID controller are also demonstrated simultaneously.

**Figure 6:**
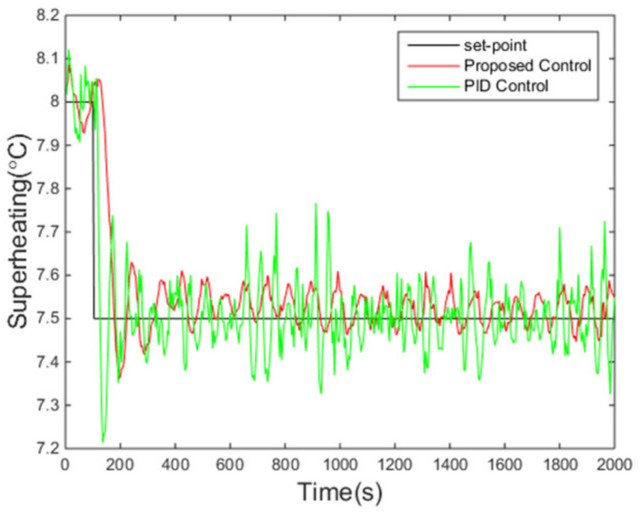
Responses of superheating.

**Figure 7:**
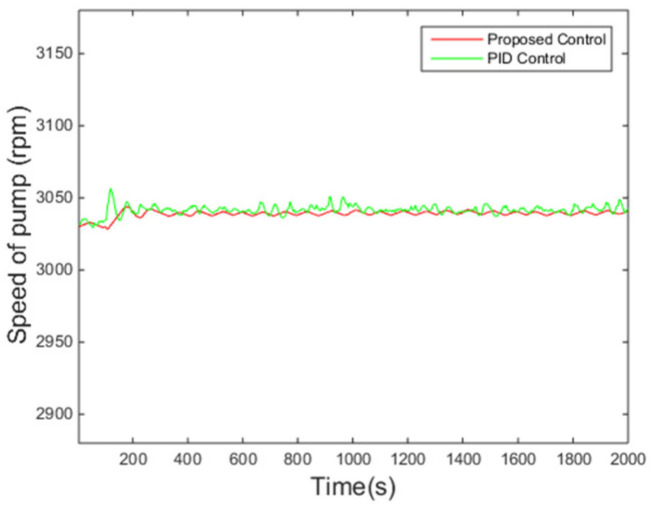
Rotating speed of the pump.

The variations of the superheating under two control algorithms are presented in Figure 6. It is evident that the overshoot under the proposed controller is smaller and the fluctuation of the superheating is also smaller when the system enters steady states compared with those under the PID control method, while the settling time is roughly the same. Meanwhile, the pump speed, the manipulated variable of the control system, is reasonable according to Figure 7.

**Figure 8:**
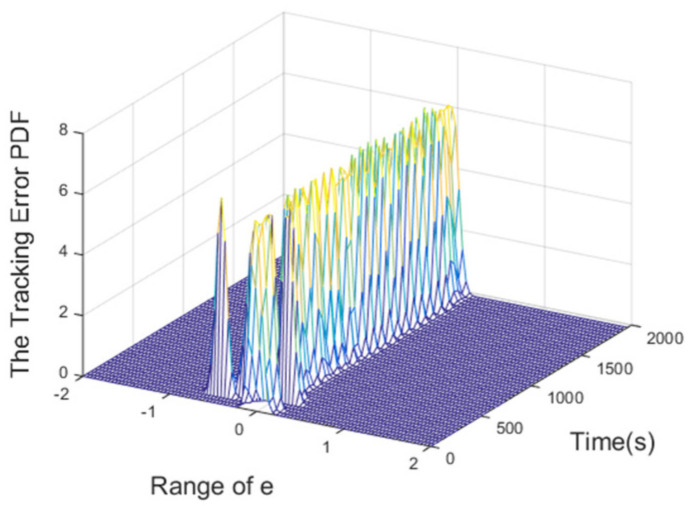
PDFs of tracking error at instants.

**Figure 9:**
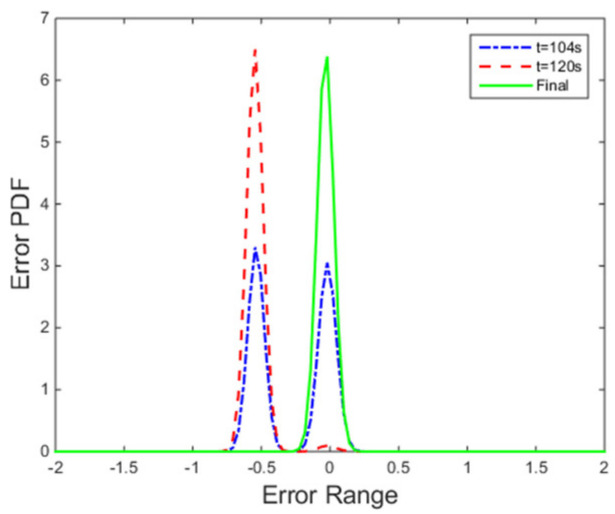
3D PDF of tracking error.

The PDFs of the tracking error at certain points are illustrated in Figure 8. Furthermore, the evolution of the PDF over time under the proposed control method is demonstrated by the 3-dimensional graph in Figure 9. The increasingly narrow shape of the PDF indicates the decreasing randomness of the superheating.

**Figure 10:**
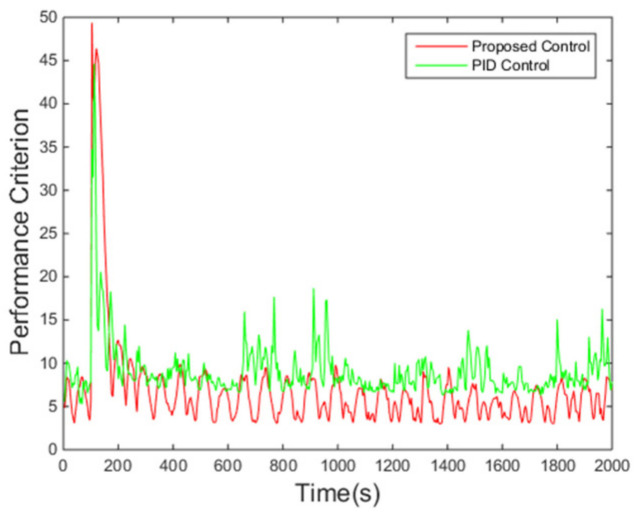
Value of the criterion.

**Figure 11:**
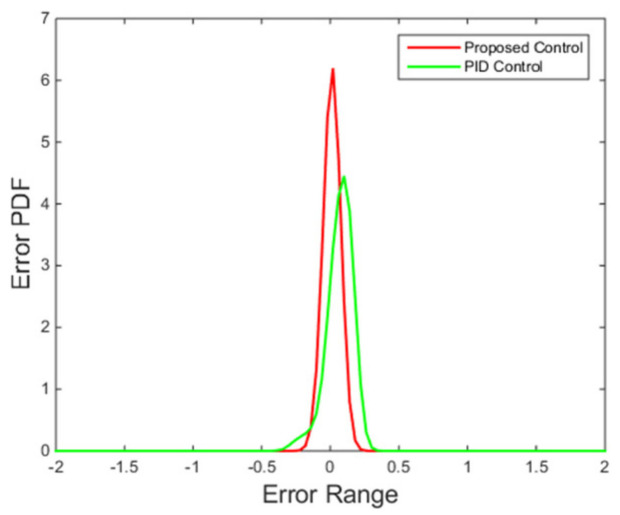
Final PDFs of tracking error.

Figure 10 shows that the performance criterion can be regulated back around a small value by the proposed control algorithm, and then fluctuates in a smaller range compared with the PID control method. The peak at around 100 s corresponds to the step change of the set point. The final PDFs shown in Figure 11 also illustrates this point with the cognition that the narrower the PDF, the smaller the randomness.

### 4.2. Disturbance Rejection

**Figure 12:**
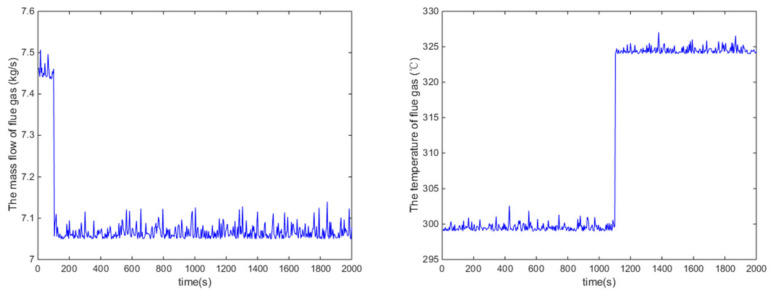
Mass flow rate and inlet temperature of the heat source over time.

In this disturbance rejection test, the mass flow rate of heat source has a 0.4 step decrease at 100 s and then the inlet temperature of heat source has a 25 °C step increase at 1100 s. The variations of the mass flow rate and inlet temperature of heat source are demonstrated in Figure 12.

**Figure 13:**
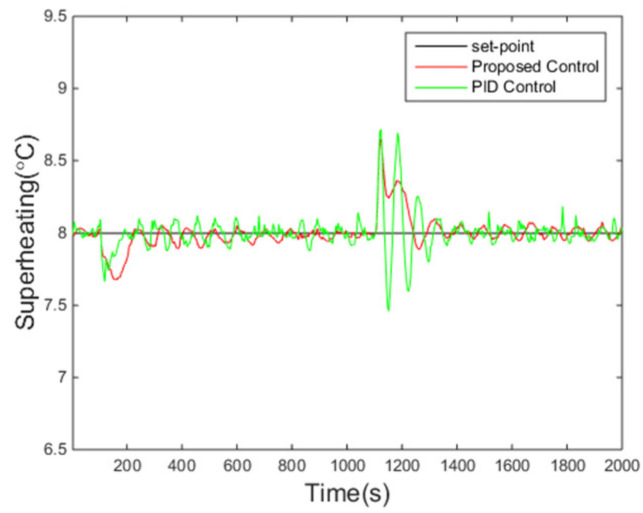
Responses of superheating.

**Figure 14:**
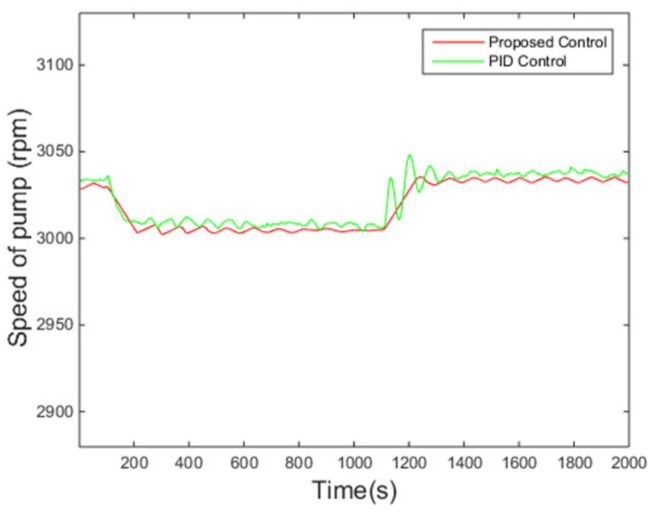
Rotating speed of the pump.

**Figure 15:**
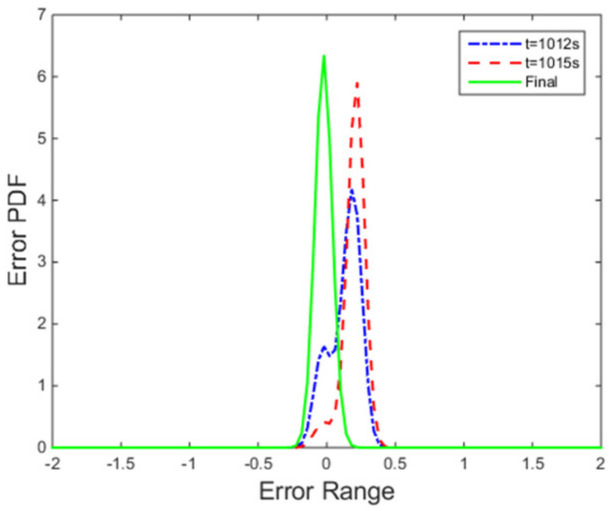
PDFs of tracking error at instants.

**Figure 16:**
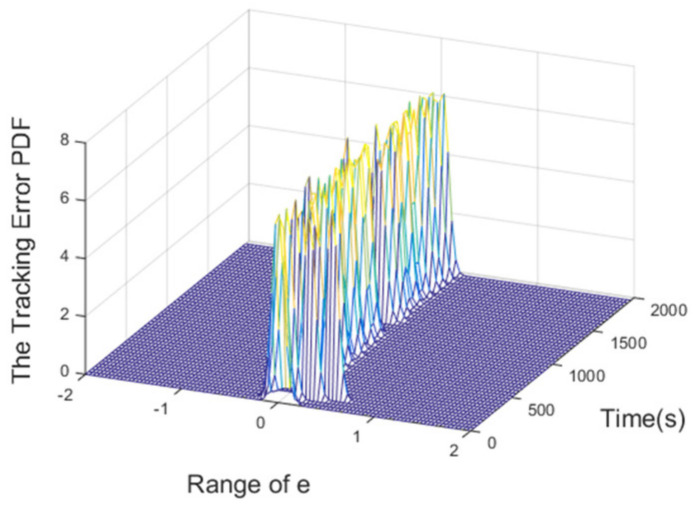
3D PDF of tracking error.

**Figure 17:**
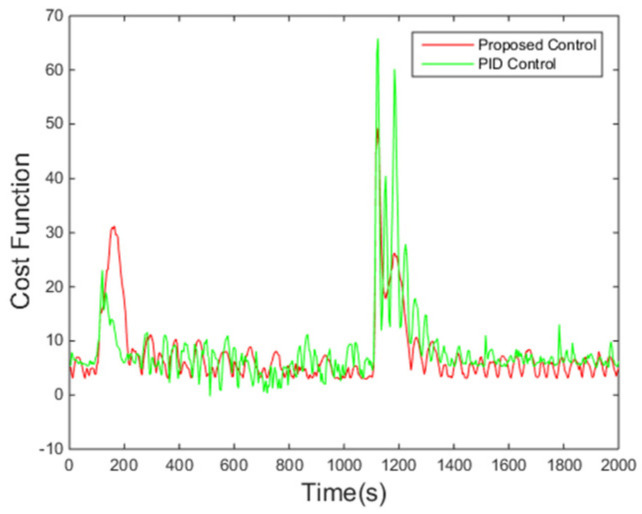
Value of the criterion.

**Figure 18:**
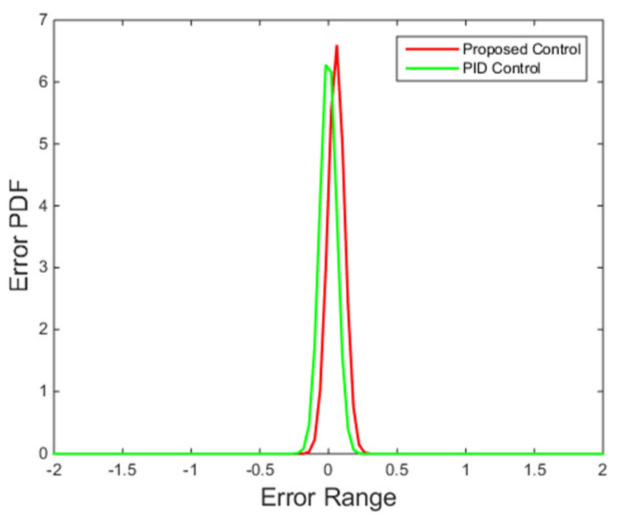
Final PDFs of tracking error.

It can be seen from Figure 13 that the proposed control algorithm has better overall performance when the mass flow rate and inlet temperature of heat source changes, and it has smaller fluctuations of superheating in the steady state compared with the PID control method. The variations of the pump speed over time are shown in Figure 14. Figure 15 and Figure 16 demonstrate that the tracking error of the superheating can be regulated to zero and then fluctuates within an acceptable small range by the proposed control algorithm after the sudden changes on the heat source. Curves of the performance criterion obtained from Equation (13) are demonstrated in Figure 17, and the two peaks correspond to the step changes of the mass flow rate and inlet temperature of heat sources which occur at 100 s and 1100 s, respectively. It is obvious that the final PDF of tracking error under the proposed control method is narrower than that of PID control as shown in Figure 18.

The simulation results demonstrate that the proposed minimum $\left(h,\varphi \right)$-entropy control algorithm is capable of achieving high performance for the ORC system subjected to non-Gaussian transient heat sources. To compare the proposed control method with the PID control more intuitively, two quantitative indicators, standard deviation (STD) and mean squared error (MSE), are presented in Table 1. The indicators of the proposed control method are smaller indicating better control performance.

## 5. Conclusions

In this work, a model-free superheating control algorithm is implemented to ORC systems with heat sources under non-Gaussian circumstances. To attenuate the randomness of the controlled ORC system and achieve accurate superheating tracking control, (*h,φ*)-entropy is adopted as the performance criterion. The superheating control law can be obtained directly by minimizing the performance criterion, and the stability analysis of the proposed control method is given. Two simulations are carried out to verify the set-point tracking and disturbance rejection ability of the proposed control algorithm compared with PID control method, and some conclusions can be drawn as follows:

1. The proposed minimum (*h*,*φ*)-entropy control algorithm is effective for the ORC system subjected transient heat sources under non-Gaussian circumstances and superheating of the ORC system can be controlled within a proper range. Moreover, the proposed control method could achieve better performance compared with PID controller in most instances;
2. Kernel density estimation (KDE) method is introduced in the proposed control algorithm for the estimating error PDF, which is used to calculate the (*h*,*φ*)-entropy of tracking error;
3. The controller design does not depend on the mathematic model of the ORC system, and the model established in this work is only for the stability analysis. The proposed control algorithm is a model-free one.

## Figures and Tables

**Table 1:** Quantitative indicators for the two controllers in the tests.

Test	Control Method	STD	MSE
Tracking Stability test	Proposed control	0.0886	0.0078
	PID control	0.0887	0.0079
Disturbance Rejection test	Proposed control	0.1278	0.0163
	PID control	0.1449	0.0210

## Data Availability

Not applicable.

## Nomenclature

A	cross sectional area (m^2^)	NN	rotating speed (rpm)
ρ	density (kg/m^3^)	ff	filling factor
$\dot{m}$	mass flow rate (kg/s)	V	volume (m^3^)
Dr	diameter (m)	v	specific volume (m^3^/kg)
P	pressure (kPa)	η	heat transfer coefficient
T	temperature (°C)	t	time
$C_{p}$	heat capacity (J/(kg. °C))	l	length coordinate (m)
L	heat exchanger length (m)	hs	enthalpy (J/kg)
$H_{\phi }^{h}$	$\left(h,\varphi \right)$-entropy	p	probability density function
$G_{\sigma }$	kernel function	σ	bandwidth
N	window width	J	performance criterion
Q	weight	R	weight
e	tracking error	T	simulation time
$T_{s}$	simulation period	N	window width
u	control input	x	state variable
ω	disturbance	k	sampling time
Subscripts			
w	wall	c	cooling water
sl	saturated liquid	pp	pump
g	saturated vapor	ra	rated
in	inlet or inner	e	evaporator
o	outlet or outer	r	working fluid
a	heat source	1	sub-cooling region
exp	expander	2	two-phase region
ss	swept	3	superheat region
